# Clinical and evolutionary features of bipolar disorder in women

**DOI:** 10.1192/j.eurpsy.2021.536

**Published:** 2021-08-13

**Authors:** M. Tfifha, W. Abbes, M. Nefoussi, M. Abbes, K. Mdhaffar, K. Zitoun, L. Ghanmi

**Affiliations:** 1 Department Of Psychiatry, regional hospital of gabes, gabes, Tunisia; 2 The Department Of Psychiatry, Hospital of gabes, Gabes, Tunisia

**Keywords:** bipolar disorder, clinical features, Gender differences, evolutionary features

## Abstract

**Introduction:**

Bipolar female patients have clinical and evolutionary features wich involve different factors related to the women specifities.

**Objectives:**

Establish clinical and evolutionary features in a population of bipolar female patients attending to Gabes psychiatry department.

**Methods:**

A retrospective descriptive and analytical study was undertaken including female patients referred to psychiatry department of Gabes regional hospital, for the first time in a 6-year period (January 1^st^, 2010 to December 31, 2016) and who were already diagnosed with bipolar disorder (BD). Sociodemographic, clinical and evolutionary data were assessed. Patients were divised into two groups according to gender. The collected data were compared between the two groups. The statistical analysis was executed on the software SPSS (20^th^ edition).

**Results:**

From the 193 bipolar patients, 103 were women. The mean age of the disorder’s onset amongst Female patients was 32.4 years old [14 - 63]. The mean duration of the disorder was 7.6 years [2 - 30]. The polarity of the first episode was a depressive one in 74.7% of cases. It was associated to psychotic features in 43.7% of cases. Seasonal pattern was noted in 10.6% amongst female patients and rapid cycling bipolar disorder in 6.2% of cases. Analytical study showed that women began the BD more often with a depressive episode (p=0.004) and were more frequently diagnosed with BD type 2 (p<0.001). Men had significantly more auditory (p=0.002) and visual hallucinations (p=0.019).

**Conclusions:**

There were clinical specifities of women with BD from which important to be considered.

